# A new species of *Curtobacterium* from apple fruit: *Curtobacterium pomorum* sp. nov. resistant to heavy metals

**DOI:** 10.1128/spectrum.02031-25

**Published:** 2026-01-06

**Authors:** Kazuki Tsuruga, Kouki Shimamoto, Kenji Okumura, Kohei Ogura, Wataru Hashimoto

**Affiliations:** 1 Laboratory of Basic and Applied Molecular Biotechnology, Division of Food Science and Biotechnology, Graduate School of Agriculture, Kyoto University 222734, Uji, Kyoto, Japan; University of Maryland Baltimore County, Baltimore, Maryland, USA

**Keywords:** apple fruit, *Curtobacterium*, genomic islands, heavy metal resistance, new species

## Abstract

The genus *Curtobacterium* has been frequently found in soil and plants. In this study, we isolated the bacterial strain designated KT1 from an apple fruit. The 16S rRNA gene sequence of the KT1 was identical to those of *Curtobacterium flaccumfaciens* and *Curtobacterium allii* type strains. The average nucleotide identities between the complete genomes of the strain KT1 and the two strains were 89.3% and 89.2%, and the digital DNA–DNA hybridization values were 64.2% and 60.5%, respectively, which were less than the threshold values for species delimitation. Phylogenetic analysis based on orthologs of genus *Curtobacterium* categorized the strain KT1 into a cluster that contained no representative or type strain genome. The strain KT1 grew at a pH range of 5.0–10.0, while *C. flaccumfaciens* JCM 1347 (=ATCC 6887) grew at pH 5.0–9.0. KT1 cells had abundant anteiso-heptadecanoic acid. The strain KT1 exclusively possessed a gene island containing genes encoding a CueP family metal-binding protein, heavy metal translocating P-type ATPases, and a metal-sensitive transcriptional regulator. Growth of the strain KT1 was significantly more than that of *C. flaccumfaciens* JCM 1347 at the concentrations of 2 µg/mL cadmium sulfate and 300 µg/mL copper sulfate, indicating that the genomic island is involved in resistance against heavy metals.

**IMPORTANCE:**

*Curtobacterium* strain KT1 showed low genome sequence identity with known species in the same genus and met the criteria for classification as a novel species. Further phenotypic and physiological characterization revealed notable differences from related species, including carbon source utilization, optimal growth temperature, pH tolerance, and fatty acid profile. Heavy metal resistance of the strain indicated its ability to grow in cadmium/copper-contaminating soils and host plants. Based on the polyphasic data, we propose that the KT1^T^ strain (=DSM 118677^T^ =JCM 37513^T^) is a novel species belonging to the genus *Curtobacterium* and name it as *Curtobacterium pomorum* sp. nov.

## INTRODUCTION

*Curtobacterium,* a Gram-positive rod-shaped bacterium belonging to the family *Microbacteriaceae*, was first isolated by Yamada and Komagata in 1972 (1) and recategorized from the genus *Corynebacterium*. At the time of writing this paper (June 2025), 11 valid scientific names have been published for the genus *Curtobacterium* in the Deutsche Sammlung von Mikroorganismen und Zellkulturen GmbH database (2). *Curtobacterium* strains are strictly aerobic and found in plant-surrounding environments, such as soil, waterweeds, grasses, and citrus leaves (3–5). As of November 2025, 13 species have been registered as validly published ones in the list of prokaryotic names with standing in nomenclature (https://lpsn.dsmz.de/genus/curtobacterium) (2), whereas 14 reference genomes were registered in the NCBI Genome database. Genomes of genus *Curtobacterium* are rich in G+C with contents of approximately 70%–72%. Previous reports have shown that genus *Curtobacterium* strains produced unsaturated menaquinones with nine isoprene units (menaquinone-9) (6, 7). *Curtobacterium flaccumfaciens* is the most extensively studied species within the genus *Curtobacterium. C. flaccumfaciens*, a phytopathogenic species causing bacterial wilt in various plants, is categorized into five pathovars, viz., *C. flaccumfaciens* pv. *flaccumfaciens* (infecting leguminous plants) (8), pv. *beticola* (infecting sugar beet) (9), pv. *poinsettiae* (infecting poinsettia) (10), pv. *oortii* (infecting tulips) (11), and pv. *ilicis* (infecting American holly) (12). In addition to *C. flaccumfaciens*, a recent study has reported that *Curtobacterium allii* 20TX0166^T^ exhibits pathogenicity to onions (13). While *Curtobacterium citreum* is known as a yellow pigment-producing bacterium, *Curtobacterium caseinilyticum*, a novel species producing yellow pigments, has been registered recently (5). In addition, Scouten et al. have reported *Curtobacterium aetherium*, which displays high levels of tolerance to desiccation and UV radiation (14).

Although *Curtobacterium* species are often isolated from soil, plant surfaces, and phyllosphere environments, their ecological functions (e.g., nutrient cycling, plant–microbe interactions, survival strategies) remain largely uncharacterized. In addition, the phylogenetic relationships among the known species are still under revision, with possible cryptic or misclassified species. In this study, we isolated a new bacterial species of the genus *Curtobacterium* from an apple fruit, termed *Curtobacterium pomorum* strain KT1, and characterized it using a polyphasic approach.

## RESULTS AND DISCUSSION

### Isolation of *Curtobacterium* strain from apple fruits

**Fig 1:**
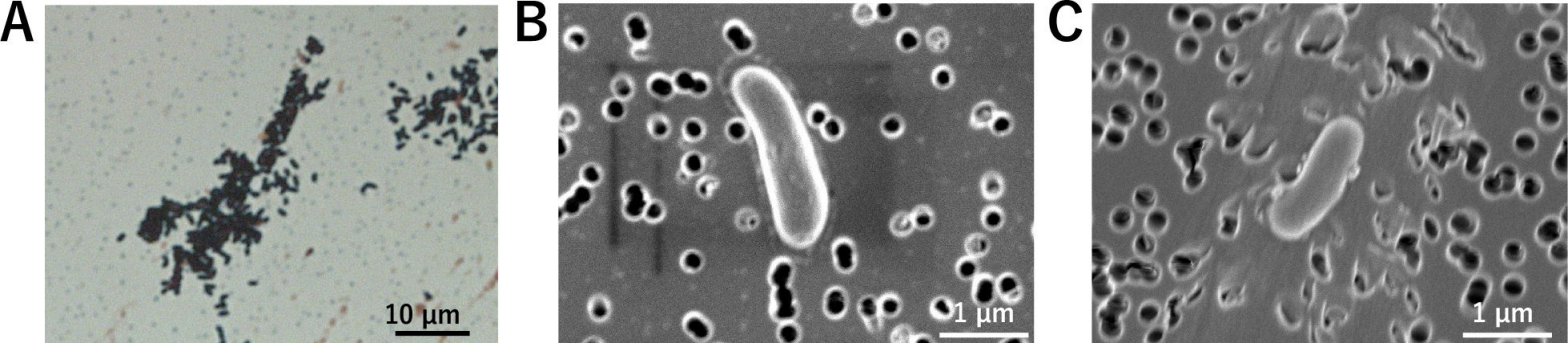
Microscopic images of the novel strain *C. pomorum* strain KT1. (**A**) Gram staining result showed that the *C. pomorum* strain KT1 cells were stained a bluish-purple color, indicating that they are Gram-positive. The scale bar measures 10 µm. (**B**) Scanning electron microscopy (SEM) observation result using NanoSuit shows that *C. pomorum* strain KT1 is a short rod-shaped bacterium without flagella. The scale bar measures 1 µm. (**C**) SEM observation result using classical fixation protocol with formaldehyde and osmium tetroxide shows the same as NanoSuit. The scale bar measures 1 µm.

Apples were mashed, squeezed, and spread onto bacterial culture plates. After incubation at 30°C aerobically for 2 days, colonies were collected for further analysis. Based on 16S rRNA sequences, one of the obtained colonies was identified as genus *Curtobacterium* and named strain KT1. Other than the *Curtobacterium* strain, we also isolated *Bacillus* sp. (species undetermined by its 16S rRNA gene sequence), *Luteibacter* sp. (species undetermined), *Pseudomonas graminis*, *Pseudomonas koreensis, Priestia aryabhattai*, *Priestia megaterium*, and *Stenotrophomonas nematodicola*. The colony of strain KT1 was yellow, rounded, and viscous. Strain KT1 was Gram-positive and non-spore-forming (Fig. 1A). The KOH test (15) also showed Gram-positive results. Strain KT1 is an irregular, short rod-shaped bacterium without flagella (Fig. 1B and C).

It is estimated that approximately 30 to 40 genes are required for complete flagellar assembly and function (16). Although the expression of flagella-related genes can vary depending on the culture conditions, the genome of this bacterium lacks the gene encoding the cap protein FliD, a key component of the flagellar structure, suggesting that this bacterium is unable to form functional flagella.

### Complete genome sequence

**Fig 2:**
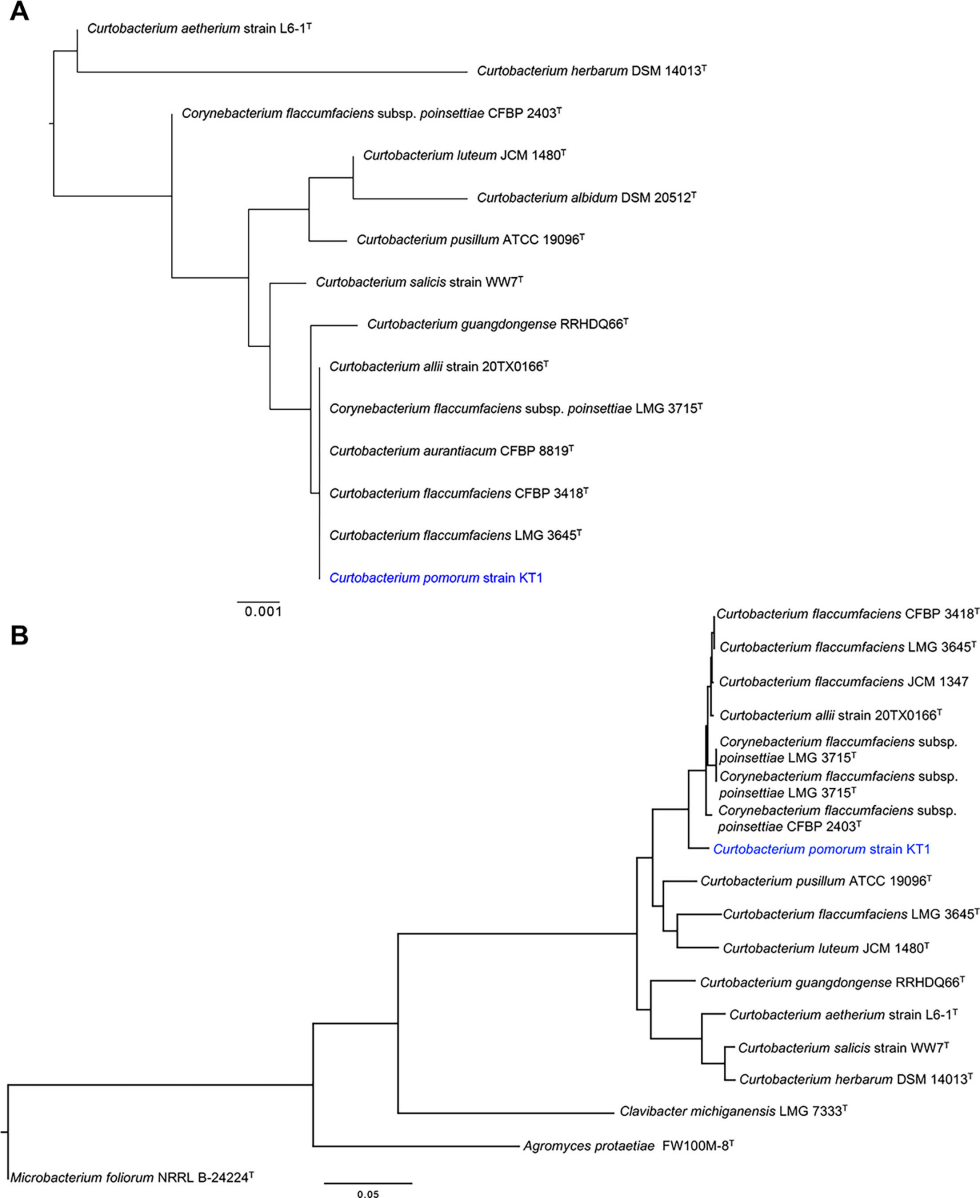
Phylogenetic analysis of strain KT1 and *Curtobacterium* type strains. The phylogenetic trees were constructed based on (**A**) 16S rRNA gene and (**B**) whole-genome sequences, and visualized using FigTree (http://tree.bio.ed.ac.uk/software/figtree/). The phylogenetic tree based on whole-genome sequences includes three outgroup species: *Agromyces protaetiae* FW100M-8^T^, *Clavibacter michiganensis* LMG 7333^T^, and *Microbacterium foliorum* NRRL B-24224^T^. Additionally, *C. flaccumfaciens* JCM 1347, which was analyzed in this study, is also part of the tree.

The isolate exhibited 100% similarity to *C. flaccumfaciens* LMG3645^T^ and *C. allii* 20TX0166^T^ based on the 16S rRNA sequences (Fig. 2A). The other species with high sequence similarity of the 16S rRNA gene were *Curtobacterium pusillum* DSM 20527^T^ (99.52%), *Curtobacterium luteum* DSM 20542^T^ (99.31%), and *C. citreum* DSM 20528^T^ (99.31%), all of which had a coverage rate of ≥99%. Using long and short reads, the complete genome of strain KT1 was determined. The genome size of the *Curtobacterium* isolate was 3,901,239 bp with a G+C content of 70.8%. Annotation using the DFAST web tool (17) detected 3,730 CDSs. Assessment of genome completeness showed 99.6% of BUSCOs classified as complete. Of these, 99.4% were complete and single-copy, while 0.2% were complete but duplicated. Additionally, 0.3% were fragmented, and only 0.1% were missing (18).

The strain KT1 was similar to *C. flaccumfaciens* CFBP 3418^T^ with an average nucleotide identity (ANI) value of 89.3% and digital DNA–DNA hybridization (dDDH) value of 64.3%, both of which were less than the threshold for the identical species (ANI ≧ 95% and dDDH ≧ 70%) (19, 20) (Table 1). The strain KT1 was also similar to *C. flaccumfaciens* LMG3645^T^ and *C. allii* strain 20TX0166^T^ with ANI values of 89.3% and 89.2%, respectively, whereas the dDDH values were 64.2% and 60.5%. These results showed that the strain KT1 is a novel species belonging to the genus *Curtobacterium*. Phylogenetic analysis using conserved bacterial marker genes (21) showed that strain KT1 is relatively close to *C. flaccumfaciens* CFBP 2403^T^ and *C. allii* strain 20TX0166^T^, yet it is situated on a distinct phylogenetic branch (Fig. 2B). Therefore, we named it as *C. pomorum* strain KT1.

**TABLE 1:** ANI and DDH values of *Curtobacterium* strain KT1 against the 13 type strains and four closely related strains’ genomes

References	Accession no.	ANI (%)	DDH (%)
Genus *Curtobacterium* type strains			
*Curtobacterium flaccumfaciens* CFBP 3418^T^	GCF_004103915.1	89.3	64.3
*Curtobacterium flaccumfaciens* LMG 3645^T^	GCF_013359815.1	89.3	64.2
*Corynebacterium flaccumfaciens* subsp. *poinsettiae* CFBP 2403^T^	GCF_018598335.1	89.2	59.7
*Curtobacterium aurantiacum* CFBP 8819^T^	GCF_018598405.1	89.2	60
*Corynebacterium flaccumfaciens* subsp. *poinsettiae* LMG 3715^T^	GCF_026241875.1	89.2	60.3
*Curtobacterium allii* strain 20TX0166^T^	GCF_021271025.1	89.2	60.5
*Curtobacterium pusillum* ATCC 19096^T^	GCF_013359865.1	86.5	49.7
*Curtobacterium albidum* DSM 20512^T^	GCF_013359825.1	85.6	43.1
*Curtobacterium guangdongense* RRHDQ66^T^	GCF_049341845.1	85.1	45.7
*Curtobacterium luteum* JCM 1480^T^	GCF_014646995.1	85.1	45.1
*Curtobacterium herbarum* DSM 14013^T^	GCF_016907335.1	84.7	38.1
*Curtobacterium aetherium* strain L6-1^T^	GCF_018885305.1	84.6	35.8
*Curtobacterium salicis* strain WW7^T^	GCF_011759505.1	84.4	38.1
Related strains			
*Curtobacterium* sp. strain JUb34	GCF_003755065.1	98.62	86.8
*Curtobacterium citreum* strain RIT_BL8	GCF_037723785.1	98.45	86.6
*Curtobacterium citreum* strain RIT_GXS8	GCF_037911255.1	98.45	86.6
*Curtobacterium* sp. strain TC1	GCF_019844075.1	97.06	72.2

### Characteristics of *C. pomorum* strain KT1

For comparison, we utilized a non-type strain *C. flaccumfaciens* JCM 1347 (=ATCC 6887 =DSM 20129), which shows ANI values of 97.6%, 97.6%, and 96.0% with *C. flaccumfaciens* LMG 3645^T^, *C. flaccumfaciens* pv. *flaccumfaciens* CFBP 3418^T^, and *C. allii* strain 20TX0166^T^, respectively. While *C. flaccumfaciens* JCM 1347 grew at pH 5.0–9.0 with pH 7.0 as the optimal condition, *C. pomorum* strain KT1 grew at pH 5.0–10.0 with the same optimal pH. Regarding temperature tolerance, *C. flaccumfaciens* JCM 1347 grew at 15°C–37°C (optimal 28°C–30°C), whereas *C. pomorum* strain KT1 grew at 10°C–35°C (optimal 28°C–30°C).

*C. pomorum* strain KT1 grew with *N*-acetyl-d-glucosamine, d-arabitol, d-aspartic acid, d-fructose, d-fucose, gelatin, glucuronamide, d-glucuronic acid, inositol, α-d-lactose, d-melibiose, d-raffinose, l-rhamnose, and d-sorbitol as nutrients but not bromosuccinic acid and citric acid (Table 2). Compared with that in five closely related species, the assimilation of carbon source was found to differ in several compounds, such as d-aspartic acid and gelatin.

**TABLE 2:** Characteristics of *C. pomorum* strain KT1 and other *Curtobacterium* strains^*a*^^,^^*b*^

Characteristics	*C. pomorum* KT1	*C. flaccumfaciens* JCM 1347 (=DSM 20129)	*C. allii* 20TX0166^T^*^d^*	*C. pusillum* DSM 20527^T^*^c^*	*C. luteum* DSM 20542^T*c*^	*C. herbarum* JCM 12140^T^ (=DSM 14013^T^)^*e*^
G+C content (mol%)	70.8	68.3	70.8	70.5	71.5	71.4
Motility	−	−	−	+	+	+
Assimilation of						
*N*-acetyl-d-glucosamine	+	+	+	+	+	−
d-arabitol	+	+	+	w	−	−
d-aspartic acid	+	−	w	N.D.	N.D.	N.D.
Bromo-succinic acid	−	w	−	−	−	N.D.
Citric acid	−	w	+	−	−	N.D.
d-fructose	+	+	+	+	+	+
d-fucose	+	−	+	N.D.	N.D.	−
l-fucose	w	+	+	−	+	−
l-galacturonic acid lactone	w	w	+	−	−	−
Gelatin	+	−	+	N.D.	N.D.	−
Glucuronamide	+	+	+	−	−	+
d-glucuronic acid	+	+	+	−	−	+
Inositol	+	+	+	−	−	+
α-d-lactose	+	+	+	−	+	+
d-mannitol	+	+	+	+	−	−
d-melibiose	+	+	+	−	+	+
d-raffinose	+	+	+	+	−	−
l-rhamnose	+	+	+	−	−	−
d-sorbitol	+	+	+	+	−	+
Tween 40	w	+	+	−	−	N.D.

^*a*^ +, Positive; w, weakly positive; −, negative; N.D., no data.

^*b*^ Data were obtained from previous reports.

^*c*^ Aizawa et al. (3).

^*d*^ Khanal et al. (13).

^*e*^ Behrendt et al. (4).

The major (>10%) cellular fatty acids of strain KT1 were anteiso-C_17:0_ (40.5%), anteiso-C_15:0_ (32.8%), iso-C_16:0_ (7.9%), and summed feature8 (C_18:1_ ω7c and/or C_18:1_ ω6c, 7.0%). The levels of omega fatty acids were higher than those of related species. Moreover, C_16:0_ fatty acids were relatively abundant (5.4%).

### Phylogenetic analysis of *Curtobacterium* genomes

**Fig 3:**
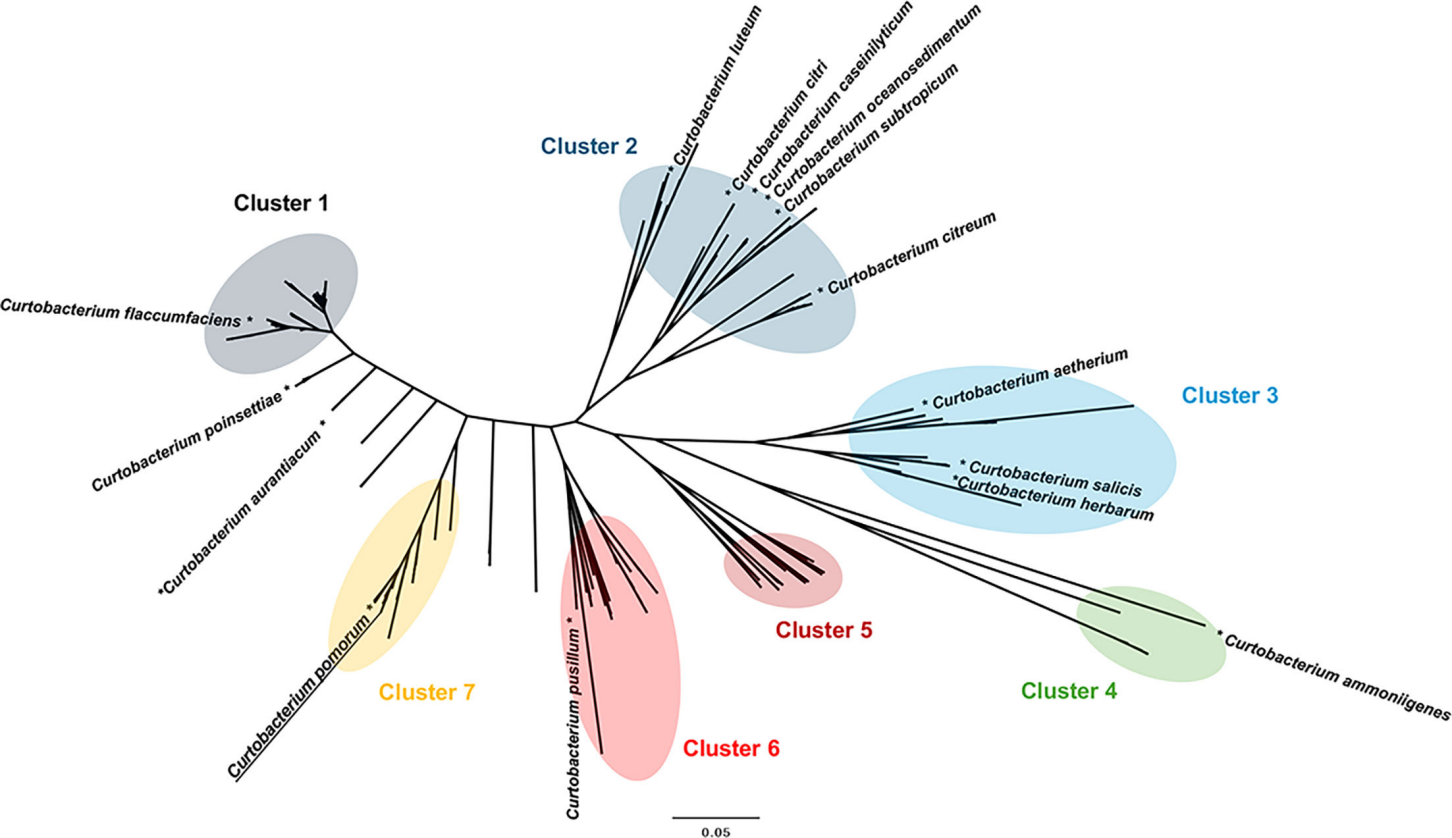
Phylogenetic trees of 334 *Curtobacterium* genomes. The tree was constructed with concatenated alignment of single-copy orthogroups detected by OrthoFinder (22). The detected clusters (1–7) were shown by colors. The tree was constructed using FigTree (http://tree.bio.ed.ac.uk/software/figtree/).

Phylogenetic diversity in the 334 *Curtobacterium* genomes was examined based on single-copy orthologs (defined as orthogroups) within the genus using OrthoFinder (22). The *Curtobacterium* genomes were categorized into seven clusters (Fig. 3). The strain KT1 and its related species were categorized into cluster 7.

Cluster 1 contained *C. flaccumfaciens* and *C. alii* strains. Consistent with a previous report (23), some strains assigned as *C. flaccumfaciens* belonged to clusters not 1 but 2, 6, and 7. Osdaghi et al. proposed that *C. flaccumfaciens* pv. *beticola* ATCC BAA-143, which is categorized into cluster 2, should be renamed as *C. citreum* pv. *basellae* based on its genome sequence (24). Evseev et al. proposed that *C. flaccumfaciens* JUb65 (cluster 6) and its related species need to be assigned to distinct species (23). *C. citreum* was also detected in various clusters: clusters 1, 2, and 7. These results showed that 16S rRNA gene sequences are insufficient for species typing in genus *Curtobacterium*.

### Closely related species

We found four isolates with ANI values of more than 95% to *C. pomorum* strain KT1: *Curtobacterium* sp. JUb34 (no information on isolated site) (98.6%), *C. citreum* strain RIT_BL8 isolated from *Marcgravia umbellata* (98.5%), *C. citreum* strain RIT_GXS8 from grape vine xylem sap (98.4%), and *Curtobacterium* sp. strain TC1 from a seed coat of *Medicago sativa* (97.1%). The genome sequences of *C. citreum* strains RIT_BL8 and RIT_GXS8 were distinct from the genome of *C. citreum* DSM 20512^T^ with ANI values of 85.6%, showing that the two strains should be typed as not *C. citreum* but *C. pomorum*.

### Flagellum-related genes

*C. pomorum* strain KT1 showed no motility on an R2A soft agar (0.25%) plate. However, this strain harbors a gene cluster comprising 16 flagellum-related genes (Fig. S1). BLAST search showed the region (1,952,051 to 1,970,065) is conserved in *C. flaccumfaciens* strains, which are motile with one to three lateral or polar flagella (25). Krimi et al. reported that *C. flaccumfaciens* strain EHF3 showed a moderate swimming and swarming motility (26). These reports indicated that *C. pomorum* strain KT1 may also exhibit motility under conditions other than those provided by the R2A medium culture plate.

### Genomic island

**Fig 4:**
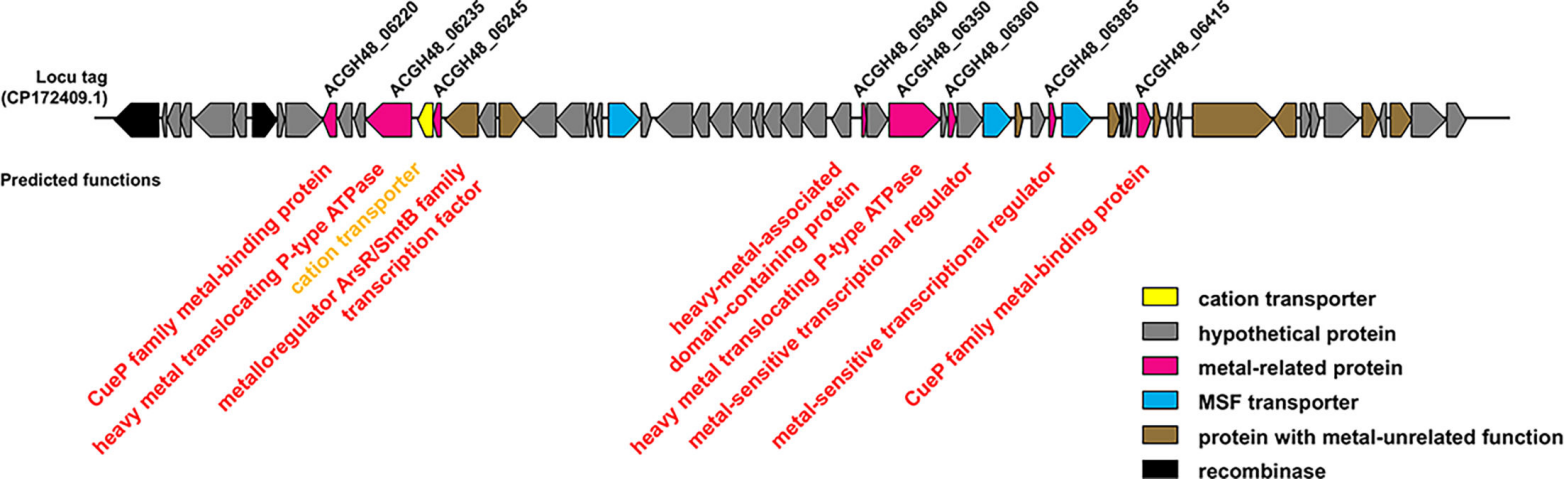
Genomic island in strain KT1 genome. Locus tags were listed in Genbank (ID CP172409.1). Functions of the encoded proteins were predicted in DFAST web tool (17). The illustration was created using the drawGeneArrows3 tool, developed by Dr. Yoshiyuki Ohtsubo from Tohoku University, Japan (https://www.ige.tohoku.ac.jp/joho/).

Islandviewer 4 (27) detected several genomic islands in *C. pomorum* strain KT1, while PHASTEST (28) showed this strain possessed no prophage region. The approximately 60 kbp genomic island spanning positions 1,283,758 to 1,344,261 was conserved exclusively in strain KT1 and absent in cluster 7 and other *Curtobacterium* species (Fig. 4) and showed partial sequence similarity with *Brevibacterium* sp., *Gordonia* sp., *Micrococcus luteus*, and related taxa. However, no significant BLAST hits were found for the full-length sequence, except in strain KT1. This island contained a gene cluster encoding heavy metal-related proteins, such as putative CueP family metal-binding proteins (Locus tags, ACGH48_06220 and ACGH48_06415 in Genbank ID CP172409.1), heavy metal translocating P-type ATPases (ACGH48_06235 and ACGH48_06350), metal-sensitive transcriptional regulator (ACGH48_06385), and metalloregulator ArsR/SmtB family transcription factor (ACGH48_06245), assuming that strain KT1 exhibits some characteristics in heavy metal transport.

### Response to heavy metal

**Fig 5:**
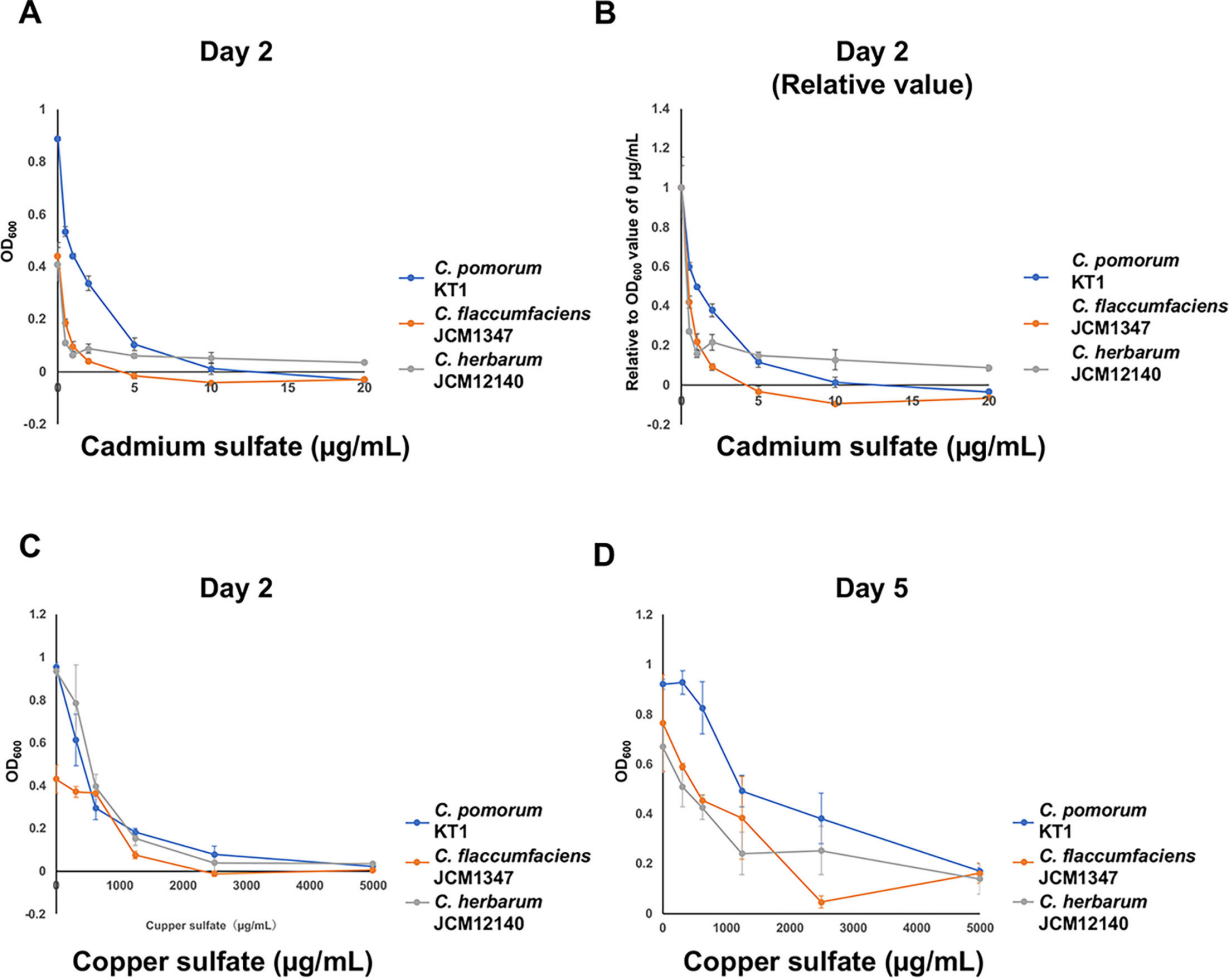
Resistance against toxic heavy metals. (**A**) Growth after 2 days of incubation in the presence of the indicated concentrations of cadmium sulfate. (**B**) Relative OD_600_ values compared to those observed without cadmium. (**C and D**) Growth after (**C**) 2 days and (**D**) 5 days of incubation in the presence of the indicated concentrations of copper sulfate.

To assess resistance to toxic heavy metals, *C. pomorum* strain KT1, *C. flaccumfaciens* JCM 1347, and *C. herbarum* JCM 12140 (DSM 14013) were incubated in the presence of cadmium sulfate or copper sulfate. The growth of *C. flaccumfaciens* JCM 1347 was inhibited at 2 µg/mL cadmium sulfate (Fig. 5A), and no growth was observed at 5 µg/mL. *C. herbarum* JCM 12140 showed growth inhibition at concentrations of 1 µg/mL or higher. *C. pomorum* strain KT1 exhibited partial growth at both 1 and 2 µg/mL cadmium sulfate.

To quantitatively compare the degree of growth inhibition, the optical density at 600 nm (OD_600_) of cultures grown without cadmium sulfate was normalized to 1, and relative growth was calculated accordingly (Fig. 5B). At concentrations of 0.5, 1.0, and 2.0 µg/mL cadmium sulfate, *C. pomorum* strain KT1 displayed significantly higher relative growth than *C. flaccumfaciens* and *C. herbarum* (Student’s *t*-test following ANOVA, *P* < 0.0167).

Next, exposure to high concentrations of copper sulfate inhibited the growth of all three *Curtobacterium* strains after 2 days of incubation (Fig. 5C). However, after 5 days, *C. pomorum* strain KT1 exhibited substantial growth at a copper sulfate concentration of 300 µg/mL (Fig. 5D). These results suggest that the genomic island present in strain KT1 may contribute to its enhanced resistance to heavy metals.

There are three types of efflux transporters involved in Cd^2+^ resistance: P-type ATPase, CBA transporters, and CDF transporter (29, 30). While strain KT1 possessed two genes encoding heavy metal translocating P-type ATPases (ACGH48_06235 and ACGH48_06350), these genes were not conserved in the other *Curtobacterium* species but in *Micrococcus luteus, Brevibacterium* sp., and *Gordonia polyisoprenivorans*. Next, in *Salmonella enterica,* CueP protein lowers copper concentrations in the cytoplasm, conferring resistance against high concentrations of copper (31, 32). Similar to P-type ATPases, *C. pomorum* strain KT1, but not other *Curtobacterium* species, possessed CueP proteins. Although the *C. pomorum* strain KT1 possessed two *cueP* genes in the island (ACGH48_06220 and ACGH48_06415), the identity of the genes was lower than the threshold of nucleotide BLAST. Identity of the two CueP proteins was moderate (44.0%). Notably, the two *cueP* genes showed no identity with the other bacterial genomes registered on the NCBI website by nucleotide BLAST. The amino acid sequence of one CueP protein (ACGH48_06220) showed identity with that of *Microbacterium* sp. (56.7% identity), the other (ACGH48_06415) resembles those found in *Kocuria* (78.8%) and *Corynebacterium* (75.9%) species. In addition, the putative metalloregulator ArsR/SmtB family transcription factor (ACGH48_06245) gene showed identity with that from *Kocuria* sp. (86.5%) and *Brevibacterium* sp. (85.3%), whereas the encoded protein showed 94.0% and 90.7% identity with Cd(II)/Pb(II)-sensing metalloregulatory transcriptional regulator CmtR from *Microbacterium* and *Arsenicicoccus* species, respectively. It is known that ArsR/SmtB family transcription factors respond to various metalloids and heavy metals, such as zinc, arsenic, and cadmium (33). Our findings indicated that the genes in strain KT1 were acquired through horizontal gene transfer from distinct bacterial lineages and subsequently mutated to acquire different functions. Further analysis is required to clarify the involvement of the genomic island in environmental adaptation.

Due to the antimicrobial properties of copper ions against bacteria and fungi by disrupting microbial cell membranes and inhibiting enzyme activity (34), copper-containing pesticides have been widely utilized in organic farming. Cadmium, a nonessential element, is toxic to host plants and microorganisms. Zhuang et al. reported accumulation of cadmium in roots, stems, and leaves of apple trees in China, while this study showed no data on apple fruits (35). Our study indicated that the acquisition of the genomic island by the strain KT1 gave an advantage for survival in environments containing these heavy metals, leading to colonization of apple fruits.

### Conclusion

In this study, we isolated a new species named *Curtobacterium pomorum*. Genus *Curtobacterium* needs to be categorized based on sequences of the whole genome, but not 16S rRNA genes, because 16S rRNA gene sequencing frequently mistypes. *Curtobacterium pomorum* strain KT1, isolated in this study, possessed a genomic island containing genes that are suggested to export toxic metals, such as cadmium and copper. The strain KT1 was registered in Leibniz Institute DSM Z (DSM 118677) and RIKEN BRC (JCM 37513).

## MATERIALS AND METHODS

### Isolation of bacterium

Apple fruits cultivated in Aomori Prefecture (Japan) were purchased from a supermarket in Kyoto Prefecture (Japan). The apple must (mashed apple) was prepared by squeezing them with a pestle in sterile bottles, followed by spreading onto bacterial culture plates containing 0.5% wt/vol glucose (Nakalai Tesque, Kyoto, Japan), 1% wt/vol tryptone (Nakalai Tesque), 1% wt/vol beef extract (Becton, Dickinson and Company, NJ, USA), 0.2% wt/vol yeast extract (Nacalai Tesque), 1% wt/vol NaCl (Nakalai Tesque), and 1.5% wt/vol agar (Nakalai Tesque), with pH adjusted to 7.2 ± 0.1. Colonies were obtained after incubating the culture plates at 30°C aerobically for 2 days. The colonies were purified by streaking them onto new culture plates.

### 16S rRNA gene sequence analysis

16S rRNA gene was amplified using the 27F and 1492R universal primers (36). Amplicons were purified using the AMPure XP Reagent (Beckman Coulter, Brea, CA, USA) and sequenced using a 3730xl DNA Analyzer (Applied Biosystems, MA, USA).

### Genome sequencing

For short-read sequencing, the genomic DNA of the *Curtobacterium* isolate was extracted using the DNeasy Blood & Tissue Kit (QIAGEN, Hilden, Germany). Short-read sequencing (paired-end 150 bp) was performed using MGI DNBSEQ (Shenzhen, China). For long-read sequencing, genomic DNA samples were extracted from the Genomic DNA Buffer Set (QIAGEN) and Genomic tip 20/G (QIAGEN), followed by sequencing using Revio (PacBio, CA, USA). Sequencing was performed at the Bioengineering Lab. Co., Ltd. (Kanagawa, Japan). Draft genome was obtained from the short-read data by *de novo* assembly SPAdes (v3.15.5) (37). Complete genome sequencing was performed using Unicycler (v0.4.1) (38) using both the short- and long-read data. The genome assembly and annotation completeness were assessed by BUSCO (v5.8.3) (18). We have confirmed that the 16S rRNA gene sequences we obtained are identical to those acquired through PCR and Sanger sequencing techniques.

### Calculation of ANI and DDH

ANI values were calculated using fastANI (39). A total of 335 *Curtobacterium* genomes were obtained from the NCBI genome database in March 2025 (Table 1; Table S1). dDDH values were calculated based on the length of all high-scoring segment pairs divided by total genome length using Genome-to-Genome Distance Calculator (GGDC 3.0) (TYGS webtool) (40) (Table 1).

### Phylogenetic analysis

GTDB-Tk was employed to construct a phylogenetic tree (Fig. 2B) that includes strain KT1, the 13 *Curtobacterium* type strains, *C. flaccumfaciens* JCM 1347, and three strains belonging to the family *Microbacteriaceae: Agromyces protaetiae* FW100M-8^T^ (GCF_004135405.1), *Clavibacter michiganensis* LMG7333^T^ (GCF_021216655.1), and *Microbacterium foliorum* NRRL B-24224^T^ (GCF_003367705.1) (21). The tree was rooted by identifying the leaf corresponding to *Microbacterium foliorum* and designating it as the outgroup. Another phylogenetic tree (Fig. 3) was constructed using OrthoFinder using 33

4 *Curtobacterium* genomes (22). The assembly data of *Curtobacterium* sp. strain S6 (GCF_000710345.2) were excluded from the analysis because of its low ANI of 76.7% with strain KT1.

### Morphology

Bartholomew and Mittwer-modified Gram staining (41) was performed using the Barmii-M kit (Muto Pure Chemical, Tokyo, Japan). Cells were treated with NanoSuit Solution Type I (NanoSuit Inc., Shizuoka, Japan) according to the manufacturer’s instructions, followed by SEM analysis using Hitachi SU8230 (Hitachi High-Tech, Tokyo, Japan). Additionally, SEM observation was performed on bacterial cells fixed using the conventional fixation protocol involving formaldehyde and osmium tetroxide.

### Growth conditions

The strain was incubated on the above-described bacterial culture plate or a Reasoner’s 2A (R2A) (0.05% wt/vol yeast extract, 0.05% proteose peptone, 0.05% casamino acid, 0.05% glucose, 0.05% soluble starch, 0.03% sodium pyruvate, 0.03% K_2_HPO_4_, 0.05% MgSO_4_·7H_2_O) agar plate under anaerobic conditions prepared by AnaeroPack (Mitsubishi Gas Chemical, Tokyo, Japan). No growth was detected on either plate. For the salt-tolerance test, the strain was incubated for 7 days at 30°C with the addition of 0%–10.0% (wt/vol) NaCl, and growth was detected in the range of 0%–9.0%.

### Carbon sources

The utilization of carbon sources was evaluated using Biolog GEN III MicroPlate according to the manufacturer’s protocol (Table 2).

### Fatty acid profiles

Strain KT1 was grown in tryptic soy broth (BD Difco), freeze-dried, and subjected to gas chromatography using the Sherlock Microbial Identification System (Version 6.2) (MIDI, DE, USA) to calculate the fatty acid composition based on the TSBA6 calculation method and TSBA6 library database (Table 3). The analysis was conducted by TechnoSuruga Laboratory Co., Ltd. (Shizuoka, Japan).

**TABLE 3:** Cellular fatty acid composition profiles of the strain KT1 and related strains^*b*^

Fatty acid	*C. pomorum* KT1	*C. flaccumfaciens* LMG 3645^*c*^	*C. allii* 20TX0166^T*d*^	*C. pusillum* DSM 20527^T*c*^	*C. luteum* DSM 20542^T*c*^	*C. herbarum* DSM 14013^T*e*^
C_14:0_	1.0	0.3	0.4	N.D.	N.D.	N.D.
Iso-C_14:0_	0.4	0.5	0.3	N.D.	3.2	0.4
Iso-C_15:0_	3.2	5.8	6.5	2.8	7.9	1.22
Anteiso-C_15:0_	32.8	48.0	52.9	19.6	39.5	43.73
C_16:0_	5.4	0.8	1.0	0.4	0.4	1.57
Iso-C_16:0_	7.9	5.4	3.5	3.9	24.0	9.5
Iso-C_17:0_	1.3	1.4	1.1	1.9	2.8	1.06
Anteiso-C_17:0_	40.5	32.1	28.5	18.6	18.4	41.43
C_18:0_	0.5	0.3	0.1	N.D.	0.4	No data
Summed feature 8^*a*^	7.0	3.6	3.8	No data	No data	No data

^*a*^ Summed feature 8 includes C_18:1_ ω7c and/or C_18:1_ ω6c.

^*b*^ Data were obtained from previous reports.

^*c*^ Aizawa et al. (3).

^*d*^ Khanal et al. (13).

^*e*^ Behrendt et al. (4).

### Heavy metal resistance

*C. pomorum* strain KT1, *C. flaccumfaciens* JCM 1347, and *C. herbarum* JCM 12140 were precultured in trypticase soy broth (TSB) (BD Bioscience) media at 30°C for 2 days, and suspended in fresh TSB media containing the indicated concentrations of cadmium sulfate (0, 0.5, 1.0, 2.0, 5.0, 10, 20 µg/mL) with the initial OD_600_ = 0.01 in 96-well plates. After incubation at 30°C for 2 days, OD_600_ was measured in TECAN Infinite M Nano (TECAN, Männedorf, Switzerland). *C. flaccumfaciens* JCM 1347 and *C. herbarum* 12140 were provided by Japan Collection of Microorganisms, RIKEN BRC, which is participating in the National BioResource Project of the Ministry of Education, Culture, Sports, Science and Technology, Japan. Since some of the OD_600_ values were below the detection limit, statistical analysis using the area under the curve could not be performed. A post hoc Student’s *t*-test was performed following the ANOVA, with a significance threshold of *P* < 0.0167 to account for multiple comparisons.

## Data Availability

Raw read data were registered with the DNA Data Bank of Japan under BioProject ID PRJDB19055 and BioSample ID SAMD00828144. The assembled genome sequence and 16S rRNA sequence were registered with the National Center for Biotechnology Information (NCBI) under accession numbers CP172409 and PQ799798, respectively. The data are available from the corresponding author (W.H.) upon reasonable request.

## SUPPLEMENTAL MATERIAL

Figure S1 spectrum.02031-25-s0001.docx Flagellum-related genes clustered in the genome of strain KT1. The illustration was created using the drawGeneArrows3 tool, developed by Dr. Yoshiyuki Ohtsubo from Tohoku University, Japan (https://www.ige.tohoku.ac.jp/joho/).

Table S1 spectrum.02031-25-s0002.xlsx List of Curtobacterium genome assembly utilized in this study.

ASM does not own the copyrights to Supplemental Material that may be linked to, or accessed through, an article. The authors have granted ASM a non-exclusive, world-wide license to publish the Supplemental Material files. Please contact the corresponding author directly for reuse.
