# Supplementary material for: A new species of Curtobacterium from apple fruit: Curtobacterium pomorum sp. nov. resistant to heavy metals
Source: Microbiol Spectr. 2026 Jan 6;14(2):e02031-25. doi: 10.1128/spectrum.02031-25 (PMC12889094; doi:10.1128/spectrum.02031-25)
Supplement: Figure S1 — Flagellum-related genes clustered in the genome of strain KT1. The illustration was created using the drawGeneArrows3 tool, developed by Dr. Yoshiyuki Ohtsubo from Tohoku University, Japan (https://www.ige.tohoku.ac.jp/joho/). [file spectrum.02031-25-s0001.docx]

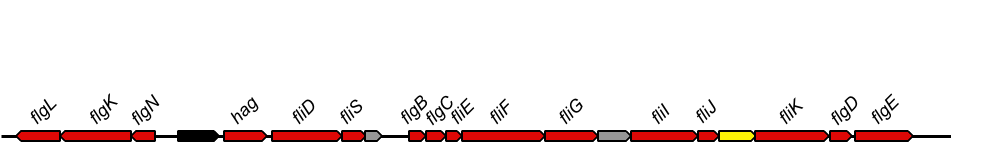


**Figure S1. Flagellum-related genes clustered in the genome of strain KT1.** The illustration was created using the drawGeneArrows3 tool, developed by Dr. Yoshiyuki Ohtsubo from Tohoku University, Japan (https://www.ige.tohoku.ac.jp/joho/).
